# Milkability in dam-calf contact systems

**DOI:** 10.1093/tas/txag040

**Published:** 2026-03-31

**Authors:** Julia Rell, Katharina A Zipp, Cornelia Buchli

**Affiliations:** Centre for Dam–Calf Contact Rearing (Fachstelle MuKa), Horgen, 8810, Switzerland; Organic Agricultural Sciences, Farm Animal Behaviour and Husbandry Section, University of Kassel, Witzenhausen, 37213, Germany; Centre for Dam–Calf Contact Rearing (Fachstelle MuKa), Horgen, 8810, Switzerland

**Keywords:** Cow-calf contact, milkability, milk ejection, nursing, oxytocin, suckling

## Abstract

Dam-calf contact (DCC) rearing is a form of cow-calf contact (CCC) in which dairy cows nurse their calves for some months until weaning while additionally being milked throughout lactation. Growing implementation, consumer demands, and research interest indicate that CCC and DCC are viable systems for the dairy industry. However, machine milking in these systems presents notable challenges with multifactorial drivers. Those are discussed in this review based on current scientific knowledge regarding milking in nursing dams: Machine milk yield (MMY), flow and fat content of the harvested milk are reduced compared to non-nursing controls while protein and lactose levels vary and udder health is unaffected or improved. In systems with full-time DCC, relative MMY reduction can reach 60–70% during the nursing period whereas highly restrictive suckling regimes cause smaller MMY losses but may not meet the calf’s nutritional needs. Post-weaning and lactation MMY can be reduced or unaffected. Low udder fills at the start of milking delay milk let-down potentially conflicting with standardized milking procedures and machine settings. A 0.5–1.5% decline in milk fat concentration indicates incomplete milk ejection and incomplete udder emptying which can further reduce MMY. These effects likely reflect a disturbance of the milk ejection reflex, as in nursing dams, suckling is a more potential stimulus for oxytocin (OT) release than milking. Underlying central mechanisms of inhibited OT release at milking in nursing dams may be related to the cow-calf bond affecting the dams’ oxytocinergic system but the complexity of required experimental set-ups compromises research in cattle. In bonded dams, multisensory stimulation by the calf may be a stronger trigger for OT release than the milking procedure. Further, separation from the calf during milking and dam-individual variation regarding the ability to release milk in response to machine stimulation may play a role. Zooming out, social scientific studies reveal that DCC farmers see poor milkability as an unsolved challenge. However, it is not limited to nursing dams, as stress affects milk ejection. To quantify incomplete udder emptying, research should refine strip milk fat analysis for on-farm use. Cow-individual milk ejection dynamics must be better understood to assess if breeding for better milk let-down in response to machine milking while maintaining good maternity traits can improve milkability in DCC systems.

## Teaser

Dam-calf contact rearing is an upcoming alternative to early separation of dairy cows and their offspring in dairy farming in the western world, but machine milk yield reduction cannot be fully attributed to calf intake. This review summarizes current knowledge on effects of nursing on milkability parameters and possible factors causing an inhibition of oxytocin release in response to machine milking in nursing dams.

## Graphical Abstract

**Figure:**
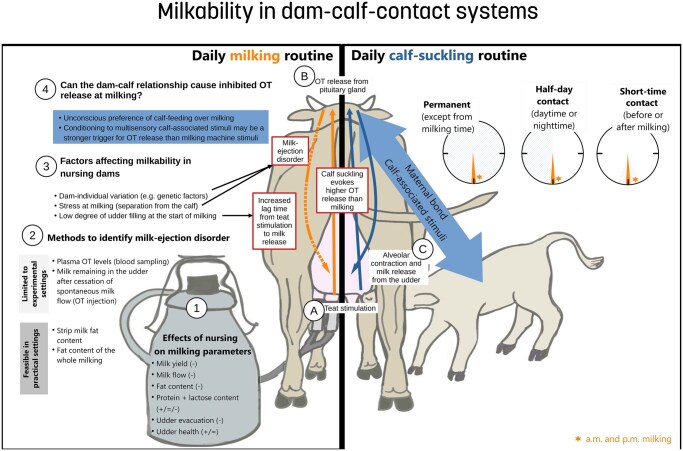

## Introduction

In commercial dairy production calves are routinely separated from their dams within the first 24 h after birth (Pempek et al. 2017). Under extensive (semi-natural) conditions cows would nurse their calves for about 7–14 months (Reinhardt and Reinhardt 1981: mean female: 9.9, male: 12.4 months; Albertsen and Held 2017: mean: 9.7 months) or until 82% of the inter-calving interval has elapsed (Albertsen and Held 2017). Even after weaning and birth of a new calf, the dam spends more time with her yearling than with other cows, which is a sign of a long-lasting social bond (Veissier et al. 1990). The cow’s machine milk yield (MMY) peaks at 40–50 days and thereafter gradually declines (Strucken et al. 2015) forcing the calf to eat roughage and thereby allowing the foregut system to develop. It must be considered that in today’s high-yielding breeds, the amount of milk produced by the dam will not restrict calf nutrition until months after peak lactation, making stimulation of solid feed intake necessary to ensure healthy foregut development.

Consumers have raised concerns regarding the early separation of cow and calf with reservations mainly referring to animal welfare issues (reviewed by Placzek et al. 2021; Sirovica et al. 2022). The most prominent reasons why early separation is so common and popular in dairy management are (1) that sellable milk is increased without dam-calf contact (DCC), (2) that cow and calf do not establish a strong bond and therefore stress-related behavior after separation is minimized and (3) that artificial rearing including controlled colostrum administration and single housing is thought to enable good passive immunity and reduce the risk of disease transmission (eg Flower and Weary 2003). Public rejection of an economically essential management factor may cause disadvantages to the dairy industry and creates demand for new approaches to cow-calf management. An increasing number of farmers are interested in cow-calf contact (CCC) rearing or start to implement CCC, mainly due to ethical considerations related to animal health and welfare (Bertelsen and Vaarst 2023; Hansen et al. 2023; Rell et al. 2024; Hautzinger et al. 2025).

Practical implementation of, as well as research on, extended CCC in dairy farming is increasing (Agenäs 2020; Eriksson et al. 2022; Cook and von Keyserlingk 2024; Whalin et al. 2025). Farmers perceive poor milkability as an additional unsolved challenge in DCC practice (Vaarst et al. 2020; Vaarst and Christiansen 2023; Rell et al. 2024). Depending on the DCC system, this can reduce MMY over the whole lactation (Barth 2020; Churakov et al. 2023; Zipp and Knierim 2024).

In this review we provide a concise summary of the current knowledge available in the scientific literature on aspects of milkability and milk let-down in nursing dams, compared to non-nursing dairy cows. The focus is on literature addressing *Bos taurus* breeds as *Bos indicus* breeds are known to require calf contact for milk let-down at milking (Orihuela 1990; Sidibe-Anago et al. 2008). Furthermore we exclude foster cow systems from this review as most dams of fostered calves are separated and milked shortly after birth conforming with early-separation systems, while nursing cows in pure foster cow-calf contact systems are, in contrast to combined rearing, often not milked during the suckling period (see Sirovnik et al. 2020). When using the term “milking” we generally refer to machine milking. “Complete udder evacuation” excludes residual milk, which physiologically remains in the udder after successful milking or suckling (Isaksson and Arnarp 1988). In the literature, milk remaining in the udder after unsuccessful udder evacuation is often, as well, referred to as residual milk (eg Negrão and Marnet 2006). In this review, we use the expression “unharvested milk” to describe this milk fraction, which includes residual milk.

## Early separation and systems of extended CCC in dairy production

Looking at dairy production from a cultural-historical point of view, early cow-calf separation is a practice that was already established in the early 20th century (Schlipf 1908). Today, it is the widespread practice in dairy cattle systems with *Bos taurus* worldwide. Calf removal within 24 h after birth (Pempek et al. 2017) is mostly followed by individual housing (60–88% in Europe, North America and Brazil; Cantor et al. 2019). Milk or milk replacer is often restricted and fed from buckets with artificial teats (Medrano-Galarza et al. 2017; Cantor et al. 2019) while weaning begins at a median age of 60 days (Gelsinger et al. 2016). Even in organic farming early cow-calf separation is the standard practice (Pempek et al. 2017).

Suckling systems have been implemented for a long time before industrial dairy production (Guintard et al. 2022) and are still used in *Bos indicus* (eg Orihuela 1990; Sidibe-Anago et al. 2008), *Bos indcus* × *Bos taurus* crossbreeds (eg Sandoval-Castro et al. 1999; Combellas et al. 2003) and in some traditional dairy systems with special breeds of *Bos taurus*, where calf presence is required for effective milk ejection (Kaskous et al. 2006; Cozma et al. 2013; Eriksson et al. 2022).

Cow-calf contact systems include DCC systems as well as foster cow systems and combined systems (Sirovnik et al. 2020). The period of CCC (contact duration) until permanent separation differs widely. The span of the median suckling period in six European countries was 5–25 weeks for female calves (Eriksson et al. 2022). Daily contact time can be unrestricted: cow and calf separation is then limited to the milking time (whole-day contact; WDC). Part-time systems include half-day contact (HDC) during daytime or nighttime and short-time contact before (CBM) or after (CAM) milking (Johnsen et al. 2016; Sirovnik et al. 2020; Eriksson et al. 2022). Cows are milked once or twice a day or with an automatic milking system (AMS; eg Johanssen et al. 2023; Ferneborg et al. 2024; Rell et al. 2024). Some farmers increase or reduce the milking frequency during the course of lactation (Rell et al. 2024) or switch from milking once daily during the nursing period to twice daily after weaning (eg Ospina Rios et al. 2023; McPherson et al. 2024). The differences in cow-calf management between early separation and DCC rearing affect physiological processes linked to milk let-down, which are mainly controlled by hormonal systems–in DCC rearing, after gestation and parturition, dams are exposed to their offspring’s presence on a daily basis, including various sensory cues and suckling. An emotional dam-calf bond is formed which is later, though at a higher age than in early separation systems (Eriksson et al. 2022; Rell et al. 2024), prematurely disrupted by artificial weaning, inducing stress (De Souza Teixeira et al. 2021). Maternal behavior and milk ejection are mainly regulated by the neuropeptide hormone oxytocin (OT), which is part of the oxytocinergic system that also contributes to stress regulation (reviewed by Bruckmaier and Blum 1998; Newberry and Swanson 2008). This makes OT a central player not only in terms of milk ejection but also as a mediator of behavioral responses linked to maternity and stress.

## Terms related to milkability

Various terms around milkability, milk ejection and situations of impairment in the milking process are used in the literature and practice. They often lack clear definitions. In the following table (Table 1) we introduce terms and definitions as used in this review including the common context of use and corresponding literature references.

**Table 1:** Terms around milkability, milk ejection and related situations of an impaired milking process as used in this review.

Term	Definition	Cause	Context/Perspective	Used or defined eg in
**Milkability**	Functional production trait for evaluation of genetic parameters that help to increase milk production but also to improve milking machines and setting parameters to achieve higher milking efficiency and a better adaptation to the physiological needs of the cow. Parameters used to determine milkability include eg MMY, milking time and milk flow characteristics.		Milk production performance; often on herd level	Schneeberger and Hagger (1985) Bruckmaier and Blum (1998) Sandrucci et al. (2007) Carlström et al. (2014) Bobic et al. (2020) Pedrosa et al. (2023) Rell et al. (2024)
**Poor/impaired milkability**	Milk removal during machine milking is slow, inefficient, or incomplete.	Physiological (eg inhibition of OT release) or anatomical dysfunction of the udder or mammary gland impeding complete udder evacuation at milking	Milking process; on individual cow level or at individual milkings	Belo et al. (2009) Rell et al. (2024)
**incomplete milk removal, incomplete udder evacuation/emptying**	More milk than the residual milk remains unharvested by the milking machine (or hand).	Reasons may be related to the cow or the milking management (eg disturbed milk ejection, inappropriate machine settings, anatomical dysfunctions)	Milking process; Milking machine or cow level	Carlström et al. (2013) Albaaj et al. (2018) Jenni et al. (2024) Rell et al. (2024)
**incomplete milk let-down, incomplete milk ejection**	failure to eject the full alveolar milk fraction into the cistern; this impedes complete milking of the alveolar fraction. Milk ejection and milk let-down are used synonymously.	The milk ejection reflex was activated but the OT level was not maintained over threshold level during the total milking duration	(Patho-)physiology of milk ejection; cow level	Bruckmaier and Blum (1998) Jenni et al. (2024) Rell et al. (2024)
**milk ejection disorder, disturbed milk ejection**	OT-induced milk ejection from the alveolar issue is hampered; this impedes complete milking of the alveolar fraction	neuroendocrine dysfunction in which central OT release in response to teat stimulation is absent, insufficient or delayed	(Patho-)physiology of milk ejection; cow level	Bruckmaier and Blum (1998) Tancin and Bruckmaier (2001) Belo et al. (2009) Jenni et al. (2024)
**milk let-down, milk ejection**	Alveolar contraction causes milk to move into the cistern in response to OT action		Physiology of milk ejection; cow level	Sagi et al. (1980) Tancin and Bruckmaier (2001) Fröberg et al. (2008)

## Physiological basics of milk ejection and udder evacuation

### Milk synthesis and storage

During lactation milk is synthesized continuously and stored in the dam’s udder. Epithelial cells of the alveolar compartment secrete milk, and capillary forces keep it fixed in the alveolar spaces and lumina of the small mammary ducts (Bruckmaier 2005). Synthesized milk first accumulates in the alveolar compartment and starts shifting to the cistern around 4 h after the last milking (Knight et al. 1994). As the cistern fills, the milk secretion rate decreases (Stelwagen 1996). Pfeilsticker et al. (1996) found that after an interval of 10–14 h between milkings, more than 80% of the milk is stored in the alveolar compartment while the cisternal milk fraction represents less than 20%. In multiparous cows the cisternal milk fraction is 2–5% greater than in primiparous cows (Pfeilsticker et al. 1996). During the course of lactation, the cisternal milk fraction decreases (eg from 17.1% to 12.3%; Pfeilsticker et al. 1996) and this decline toward late lactation is more prominent in older than in younger cows (Pfeilsticker et al. 1996). Caja et al. (2004) also found a significant decrease in both alveolar and cisternal milk volumes as lactation advanced but in contrast to the study results of Pfeilsticker et al. (1996), the proportion of cisternal milk did not change in a linear pattern: cisternal milk accounted for 33% of the total stored milk in early lactation, decreased to 23% in midlactation, and then increased again to 43% in late lactation due to a comparatively greater reduction in alveolar volume (Caja et al. 2004). However, because cisternal milk in Caja et al. (2004) was determined after udder manipulation by sonography and without verification of basal oxytocin concentrations, partial milk ejection cannot be excluded and cisternal fractions may therefore have been overestimated. Cisternal milk can be removed from the udder without activation of the milk ejection reflex, while alveolar milk needs to be actively expelled into the cistern (reviewed by Bruckmaier 2005).

### Milk ejection

Under natural conditions, the neuroendocrine milk ejection reflex serves to regulate the release of milk from the maternal udder in response to the offspring’s innate behavior of teat stimulation. Oxytocin is synthesized in the supraoptic and paraventricular nuclei of the hypothalamus. From there it is transported along the axons of the OT-producing neurons to the posterior pituitary where it is stored until release into peripheral blood circulation. In response to tactile stimulation of receptors on the teat skin, nerve signals are carried via somatic sensory nerves to the hypothalamus, transported to the posterior pituitary, where OT is released into the vascular system and transported via the blood circulation to the udder. Upon docking to specific OT receptors on the myoepithelial cells surrounding the alveoli and smaller milk ducts a contraction is evoked and stored milk shifts to the cisternal space (reviewed by Svennersten-Sjaunja and Olsson 2005). Schams et al. (1984) showed that the magnitude of OT release is very variable even within individual animals whereas milk ejection response is highly repeatable. This led to the conclusion that an elevation of plasma OT concentration to an individual threshold level beyond the permanent baseline level is sufficient to evoke complete milk ejection. The existence of this threshold was confirmed by OT infusion experiments (Bruckmaier et al. 1994), ie OT must be released to induce maximum milk ejection, whereas its amplitude does not matter. However, OT must remain elevated throughout milk removal by calf or milking machine. Because of the short half-life of 2–3 min (Belo and Bruckmaier 2010) complete udder emptying requires continuous release of OT (Bruckmaier et al. 1994). Simultaneously with the release of OT into circulation, OT is also released in different brain regions which is the basis for establishing and enforcing the social bond between the dam and her calf at each suckling event (see chapter 9.1.).

### Udder evacuation through suckling versus milking

In the literature a span of 1–9 suckling bouts per 24 h with a mean duration of 3–17 minutes per bout is reported in calves with access to their dams. With increasing age, bout duration increases while the number of bouts decreases (Walker 1962; Ewbank 1969; Somerville and Lowman 1979; Reinhardt and Reinhardt 1981; Vitale et al. 1986, Lidfors et al. 2010). However, this seems to depend on management aspects, as Wegner et al. (2025) found this behavioral change in calves in a cow-driven DCC system but not in a calf-driven DCC system.

Calves’ suckling technique differs from mechanical milking. According to Rasmussen and Mayntz (1998), calves generate a mouth vacuum of 35–40 kPa during suckling, supplemented by teat compression between the tongue and palate, which leads to positive pressure in the teat cistern of 35–40 kPa. They observed two vacuum peaks per second, with pressure near zero between them; swallowing likely occurred during some, but not necessarily all, vacuum drops. An early study found that the differential pressure measured in the teat canal is lower during milking compared to suckling (McDonald and Witzel 1966: 44.2 kPa versus 71.3 kPa). Overall, the mechanical stress on the teat is higher during milking than during calf suckling, as a continuous vacuum acts on the teat in addition to cyclic liner compression during milking (Odorčić et al. 2019). Additionally, in DCC the cow is suckled by only one calf: thus the tissue of three teats can recover, while one teat is suckled. These differences in vacuum dynamics, mechanical force, and teat load show that suckling and milking represent fundamentally different forms of udder stimulation.

## Etiology of milk ejection disorders and impaired milkability

### Disturbances of milk ejection and milk removal

In addition to external factors such as unsuitable milking machine settings or udder preparation techniques, milk removal can be negatively affected by dysfunctions of the udder (eg teat injuries) and mammary gland (milk ejection disorder). A distinction must be made between poor (or impaired) milkability and disturbed milk ejection or milk ejection disorder (see Table 1). The latter specifically refers to a dysfunction of the neuroendocrine reflex, defined as central inhibition, meaning that the amount of OT released from the posterior pituitary is below an individual threshold level which must be reached to evoke milk ejection (Bruckmaier et al. 1992; Bruckmaier et al. 1993; Belo et al. 2009). In this case, no milk ejection is evoked, while incomplete milk ejection occurs if the milk ejection reflex is activated but OT levels do not persist over threshold level during the total milking duration (Bruckmaier et al. 1994; discussed in Rell et al. 2024). On the level of the mammary gland (peripheral inhibition), blockade of milk ejection is limited to experimental conditions by postsynaptic alpha-receptor stimulation or OT receptor blockade as it was used in various experimental settings (Bruckmaier 2005; Jenni et al. 2024). In contrast, the term “poor/impaired milkability” includes anatomical dysfunctions of the udder for example due to recent or healed teat injuries or mastitis which can decrease milk flow and MMY (Querengässer et al. 2002) but do not affect OT release from the brain in the first instance, though OT release might be inhibited as a consequence of pain.

### Factors affecting milk ejection in non-nursing cows

In addition to DCC-specific aspects that only nursing cows are exposed to (see chapters 8. and 9.), it is important to consider that a number of circumstances which are not related to nursing can negatively affect milk let-down at milking.

#### Early phase of the first lactation

Published research in non-nursing dairy cows suggests that primiparous cows in the postpartum phase are particularly susceptible to disturbed milk let-down. Bruckmaier et al. (1992) assumed that 1% of primiparous cows in Switzerland are affected by disturbed milk ejection while Kraetzl et al. (2001) report the figure of 10% of parturient primiparous cows affected. In Saxony, Germany, analysis of 271 questionnaires revealed that 24% of the farm managers rated more than 10% of their primiparous animals were affected (Geidel 2005; cited by Heidig 2007). However, in many studies performed in primiparous non-nursing animals no comparison was made with multiparous cows (Bruckmaier et al. 1992; Van Reenen et al. 2002; Heidig et al. 2011). One study in which parity was considered as a potential risk factor for disturbed milk ejection found no differences in its incidence between primiparous and multiparous non-nursing dairy cows (Belo et al. 2009). As most of the previously cited studies are more than 20 years old, their results should be confirmed by more recent research.

#### Unfamiliar milking environment

Bruckmaier et al. (1993) found that in multiparous cows relocated to an unfamiliar surrounding for milking, only the cisternal fraction (9% of total milk) could be removed by normal milking while OT release failed. Riedl et al. (2001) found that only 30% of total MMY was harvested in non-nursing cows after transport and admission to an animal clinic. Transient phases of disturbed milk ejection may therefore be attributed to stressful management changes cows need to adapt to (Bruckmaier et al. 1996).

#### Individual animal characteristics

In an experiment by Van Reenen et al. (2002) individual differences in the behavioral and physiological responses to milking were investigated in non-nursing primiparous cows. Indicators of milk ejection disorder (high levels of unharvested milk, low peak milk flow rates, low MMY and low OT levels) and increased heart rates during udder preparation were found to be consistent at the animal level meaning that stable individual characteristics–especially those related to stress perception or the ability to cope with stress, such as fearfulness–can mediate the physiological response to milking. Heidig (2007) found more cases of disturbed milk let-down in primiparous cows with a low stress resistance (individual response of heart rate, electromyogram, skin conduction, electrical skin resistance and behavioral response to a standardized set of stressors), were low ranking in the herd or were introverted (timid, humble) in a behavior test. In accordance with this, fearful reaction to novel situations in dairy goats was associated with more milk ejection disorders (Lyons 1989). Sutherland et al. (2012) found an impact of exit time from a restraint device (used as an indicator of temperament) on MMY in a novel environment, but no differences in milk flow, OT level after milking and unharvested milk between cows of different temperament categories. In farm animal research it must be considered that the used terms related to personality and character are not based on standardized definitions (Finkemeier et al. 2018) which adds a certain vagueness to the results of such studies. In breastfeeding women, individual anxiety is associated with to reduced OT release in response to breastfeeding (Uvnäs-Moberg et al. 1990).

It should be considered that, next to inherited traits, physiological reactions to stress depend on individual experiences. For example, negative experiences with the milking environment–such as stray voltage in the parlor (reviewed by Reinemann 2012)—or with the milking staff can influence milk let-down. Negative interactions directed by the milker toward the cow in the parlor were negatively correlated with MMY in epidemiological studies (Hemsworth et al. 2000; Waiblinger et al. 2002). In one experiment, the presence of a person who previously handled the cow aversively, in the parlor increased the amount of unharvested milk by 70% (Rushen et al. 1999).

#### Stress as the underlying trigger of inhibited OT release

All three previously described factors are fundamentally connected to stress: the experience of first parturition, the introduction to the cow herd and the first milkings represent a transient stressful phase in which the prevalence of milk ejection disorders in non-nursing primiparous cows is likely to increase. Different mechanisms possibly mediating the inhibition of OT release due to stress have been discussed (reviewed by Tancin and Bruckmaier 2001). In Bruckmaier et al. (1993), disturbed milk let-down was accompanied by increased concentrations of cortisol and beta-endorphin. The suitability of cortisol as a stress marker is limited due to ultradian and circadian variation (Lefcourt et al. 1993; Godoy et al. 2018) and physiologically increased levels during milking (Gorewit et al. 1992; Van Reenen et al. 2002). However, an inhibitory effect of cortisol on OT release is unlikely as intravenous administration of cortisol had no effect on milk ejection in cows (Mayer and Lefcourt 1987).

Beta-endorphin is released in response to activation of the hypothalamic-pituitary-adrenal axis and binds to opioid receptors reducing stress and pain perception (Pilozzi et al. 2020). Exogenous morphine used as opioid model in cows inhibits OT release (Tancin et al. 2000) and MMY and OT levels increase gradually with a concomitant decrease in β-endorphin and cortisol concentrations as cows accustom to a new location (Bruckmaier et al. 1996). Although the exact mechanisms are not yet fully understood, endogenous opioids are likely involved in central blockade of OT release (Wellnitz et al. 1997; Macuhová et al. 2002).

Noradrenaline and adrenaline, released under stressful conditions, do affect oxytocinergic neurons via α1-adrenergic receptors and β-adrenergic receptors respectively. However, adrenaline administration affected OT release during milking in both directions (reviewed by Tancin and Bruckmaier 2001) and, under stressful conditions, administration of a β-adrenergic antagonist did not abolish the central inhibition of OT release (Wellnitz et al. 1997). More research is needed to clarify the effects of stress-induced catecholamine release on milk ejection in dairy cows and to identify the mechanisms underlying stress-induced inhibition of OT release from the pituitary gland.

In nursing cows, the dam-calf bond must be considered when discussing emotional stress and its effects on behavior during milking and milk let-down (see chapter 9.).

## Effects of nursing on milking characteristics

Along with the growing number of published studies investigating different DCC systems, some effects of DCC on milking characteristics have been demonstrated.

### Machine milk yield

During the nursing period MMY is reduced in suckled cows in comparison to non-nursing cows. Table 2 shows results of various studies comparing MMY of nursing cows kept in different DCC systems to MMY of a non-nursing control group. Relative MMY reductions of more than 60% were found in most studies with WDC systems (Johanssen et al. 2024; Neave et al. 2024a; Zipp and Knierim 2024) and in one study group with CBM (Barth 2020). In all study groups with WDC, daytime contact, nighttime contact and CBM systems MMY reductions were significant (or tendencies) compared to a control group without calf contact, while among CAM groups relative MMY reductions were not significant in some cases (Cozma et al. 2013; Rell et al. 2024). Where milking times have been analyzed separately in daytime contact systems, morning milking yield–following overnight separation from the calf–did not differ from that of a control group (Neave et al. 2024a; Zipp and Knierim 2024).

**Table 2:** Overview of study results (SR) demonstrating significantly reduced (-) or unaffected (=) machine milk yield and/or the relative change of milk yield (RC) (in relation to yields of non-nursing control cows in the respective study) in nursing cows kept in whole-day contact, half-day contact or short-time contact (before milking (CBM) or after milking (CAM)) compared to a non-nursing control group.

phase	SR	RC(%)	Whole-day contact	n	CD (wk)	SR	RC(%)	Half-day contact	type HDC	n	CD (wk)	SR	RC(%)	Short-time contact	CBM	CAM	n	CD (wk)
**pre-weaning**	–	68,9	Neave et al. (2024a) ^a^	24	8–9	–	57,1	McPherson et al. (2024) ^h^	Nighttime	18	8	–	71,4	Barth (2020)	x		15	13–14
	–	67,8	Johanssen et al. (2024) ^b^	10	6	–	52,8	Bryant et al. (2024) ^h^ ^,^ ^o^	Night-/Daytime	15	9	–	51,2	Nicolao et al. (2022) ^j^	x		14	12
	–	62,0	Zipp and Knierim (2024)	13	9	=/-	45,0	Zipp and Knierim (2024) ^i^	Daytime	11	9	–	44,1	Rell et al. (2024) ^e^	x		55	3–24
	–	61,5	Zipp et al. (2018) ^c^	15	13	–	43,2	Barth (2020)	Nighttime	18	13–14	–	43,6	Boden and Leaver (1994) ^m^		x	8	31
	–	60,1	Van Zyl et al. (2025) ^d^	10	16	–	42,4	Nicolao et al. (2022) ^j^	Daytime	14	10	–	37,4	Krohn (2001)		x	19	6–8
	–	56,1	Barth (2020)	54	13–14	–	36,6	Johnsen et al. (2015)	Nighttime	10	6	–	28,7	Nicolao et al. (2022) ^j^ ^,^ ^l^		x	14	12
	–	44,1	Rell et al. (2024) ^e^	87	3–48	–	35,5	Ospina Rios et al. (2023) ^m^	Daytime	16	10	–	27,8	Mendoza et al. (2010) ^p^		x	16	8
	–	43,9	Churakov et al. (2023) ^f^	17	16	=/-	30,0	Neave et al. (2024a)^a^^,^^i^	Daytime	23	8–9	–	26,5	De Passillé et al. (2008)		x	10	9
	–	43,3	McPherson et al. (2024)	14	8	–	43,2	Van Zyl et al. (2025) ^r^	Daytime	12	16	=	13,9	Cozma et al. (2013) ^k^		x	6	6
	–	43,1	Krohn (2001)	20	12							=	2,3	Rell et al. (2024) ^e^		x	40	12–28
	–	41,9	Wenker et al. 2022b	20	7													
	–	36,4	Van Zyl et al. (2025) ^g^	9	16													
**post-weaning**	–	27,5	Johanssen et al. (2024)	10	6 (10–11)	–	22,3	McPherson et al. (2024)	Nighttime	18	8 (9–35)	–	25,0	Nicolao et al. (2022)j	x		14	12 (14–16)
	–	26,9	Van Zyl et al. (2025) ^d^ ^,^ ^s^	10	18 (19)	–	18,6	Van Zyl et al. (2025) ^s^	Daytime	11	16 (17)	–	24,2	Barth (2020)	x		15	13–14 (14–29)
	–	17,8	McPherson et al. (2024)	14	8 (9–35)	=	10,6	Bryant et al. (2024) ^h^	Night-/Daytime	15	9 (9–41)	=	2,0	De Passillé et al. (2008)		x	10	9 (10–15)
	–	15,6	Zipp and Knierim (2024)	13	9 (11–12)	=	7,2	Zipp and Knierim (2024)	Daytime	11	9 (11–12)	=	+1,4	Krohn (2001)		x	19	6–8 (w-36)^t^
	–	15,2	Barth (2020)	50	13–14 (14–29)	=	+2,6	Nicolao et al. (2022) ^j^	Daytime	14	10 (14–16)	=	N.A.	Mendoza et al. (2010) ^p^		x	16	8 (11)
	=	N.A.	Krohn (2001)	20	12 (13–24)	=	+3,9	Ospina Rios et al. (2023) ^m^	Daytime	16	10 (11–28)							
	=	9,0	Van Zyl et al. (2025) ^g^ ^,^ ^s^	9	18 (19)	=	+6,1	Barth (2020)	Nighttime	18	13–14 (14–29)							
						=	N.A.	Johnsen et al. (2015)	Nighttime	10	6 (7–21)							
**whole lactation**	N.A.	33,4	Barth (2020) ^q^	48	13–14	–	31,8	McPherson et al. (2024)	Nighttime	18	8	N.A.	45,6	Barth (2020) ^q^	x		14	13–14
	–	24,2	McPherson et al. (2024)	14	8	N.A.	20,6	Bryant et al. (2024) ^h^	Night-/Daytime	15	9	=	6,3	Margerison et al. (2002) ^n^		x	12	26
	–	23,8	Zipp and Knierim (2024)	11	9	N.A.	11,3	Barth (2020) ^q^	Nighttime	17	13–14							
						=	9,7	Zipp and Knierim (2024)	Daytime	10	9							
						–	8,0	Ospina Rios et al. (2023) ^m^	Daytime	16	10							
						=	N.A.	Johnsen et al. (2015)	Nighttime	10	6							

Contact durations (CD) until weaning are given in weeks with the period considered for calculation of the post-weaning milk yield in brackets. N represents the number of nursing cows in the respective study group. Studies are sorted from highest RR to lowest RR.

a Milk yield measured at eight milkings per cow in week four and six of lactation; two days per week (morning and evening milking).

b Six weeks whole-day contact plus two weeks part-time contact (gradual weaning); Data refer to week 1–6.

c Milk yield was measured during the second month of life.

d Experiment 2; Group “nose flap weaning before separation”.

e Cross-sectional study on four farms with whole-day contact, three farms with contact before milking and three farms with contact after milking; Two milkings considered; Milkyield per milking, not per day.

f Cows could leave the contact area voluntarily.

g Experiment 2; Group “nose flap and fenceline weaning before complete separation”.

h Cows milked only once a day until week six and afterwards twice a day.

i No difference in the morning milking; Lower milk yield in the evening milking (after daytime contact).

j Results until week eight (when all cows nursed their calves).

k Only results of the Prim Holstein groups considered; Calf suckling for one minute before milking; Calf presence during milking; Calf suckling after milking until the udder was empty.

l Experiment prematurely stopped in week eight due to low calf weight gains.

m Cows only milked once a day.

n Experiment 1 only own calf-group compared to artificially reared group.

o Fifteen hour-contact from 4.00 pm–7.00 am and milking once a day at 3 pm until week six; thereafter twice a day milking and daytime contact until weaning.

p Milk yield during week two to eleven (=incl. post weaning); no significant difference in week eleven.

q Values based on the weighted average of daily milk yields of three given phases of lactation (≤100 day, 101–200 days, 201–305 days).

r Experiment 1; Sampling period during one week after change from whole-day contact (first eight weeks of life) to daytime-contact (week nine to sixteen).

s Sampling period during one week after separation.

t w = weaning.

Post-weaning effects on MMY were mostly found in WDC systems (Barth 2020; Johanssen et al. 2024; McPherson et al. 2024; Zipp and Knierim 2024), but also in one study group with nighttime contact (McPherson et al. 2024) and two study groups with CBM (Barth 2020; Nicolao et al. 2022). It must be considered that definitions of post-weaning phases differed widely–ranging from two weeks post-weaning to the end of lactation–which requires caution when comparing results. In summary, lactation MMY of nursing dams versus non-nursing controls can be reduced or unaffected, while relative MMY reductions during the nursing period can reach more than 50% in nearly all contact systems except CAM where MMY reductions are lower (Table 2).

The main reason for reduced MMY in nursing cows is calf intake. Milk intake during suckling has been assessed by the weigh-suckle-weigh method in different short-time contact systems, with daily intakes ranging from 7 to 12 L (Boden and Leaver 1994; Fröberg et al. 2008; De Passillé et al. 2008; Mendoza et al. 2010; Bieber et al. 2022; Nicolao et al. 2022). An exception are highly restrictive systems or systems with DCC during (Lucht 2009: 4.6 kg/d) or after milking where calf intake can be substantially lower, creating a risk of undernourishment (Margerison et al. 2002: 1.1 kg/d; Nicolao et al. 2022: 3.0 kg/d). More frequent udder evacuation by additional suckling could be expected to stimulate milk secretion and increase total milk production (suckled plus milked milk), as seen after introduction of AMS (Svennersten-Sjaunja and Pettersson 2008), increased milking frequency in the parlor (e.g., Bar-Peled et al. 1995; Hale et al. 2003; Eslamizad et al. 2010), or foster rearing with full-time contact to two to four calves without milking (e.g., Everitt and Phillips 1971; Peel et al. 1979). This, however, has been found rarely, for example in the study of Bar-Peled et al. (1995), where dams were milked and suckled (by two calves) each three times a day. In contrast, in most studies, especially with WDC, differences in MMY between the nursing group and a non-nursing control group are approximately 15–20 kg/d (Zipp et al. 2018; Nicolao et al. 2022; Mac et al. 2023; Neave et al. 2024a). This indicates that calves with WDC can suckle more milk than in restricted systems, where the weigh-suckling-weigh method is possible (references above). However, it has been suggested that calves may consume similar amounts of milk during a restricted contact time, as calves’ weight gains with CBM (Roth et al. 2009) or daytime contact were comparable to those with WDC (Zipp and Knierim 2024). Milk intake in half-day or WDC systems could in principle be assessed by adding deuterium oxide to the drinking water of dams. This method has been used in other species (reviewed by Riek et al. 2007) and cattle (eg Andrew et al. 1995), though not yet in the context of milk intake in DCC. However, variation in milk intake among calves can be substantial, and methods such as deuterium oxide dilution or weigh-suckle-weigh may be stressful, interfere with cow–calf interactions, and are further complicated by allosuckling, which can make their application unfeasible in DCC systems (cited from Churakov et al. 2023). Furthermore, in some studies, existing MMY reductions during the nursing phase cannot be fully explained by the ingested amount of milk (Nicolao et al. 2022; Neave et al. 2024a).

Reducing the daily DCC time does not necessarily lead to lower ingested milk amounts (De Passillé et al. 2008) or time spent suckling (Jensen et al. 2024). However, a slight increase in MMY could be achieved by switching from WDC to HDC (Van Zyl et al. 2025; Vogt et al. 2025). When calves could not suckle due to nose-flaps or fence-line separation, their dams gave more milk compared to WDC (Van Zyl et al. 2025; Vogt et al. 2025) or gradual separation (Vogt et al. 2025). Vogt et al. (2025) compared dams’ responses to two separation methods after three months of cow-calf contact: (1) a two-step method, where calves had two weeks of full-time contact while wearing nose-flaps, followed by one week of fence-line contact before total separation; and (2) gradual reduction of contact time (one week of HDC, followed by one week of morning contact and one week of fence-line contact before total separation). During the first week of nose-flap-weaning the MMY of dams was about 3.5 kg/d lower compared to the second and third week, which might indicate an influence of weaning distress (increased vocalization and searching behavior, no change in rumination or lying: Vogt et al. 2025). Gradual reduction over three weeks of DCC, however, led to even more vocalization and searching behavior compared to noseflap-weaning (Vogt et al. 2025). Dams of the nose-flap treatment tended to have a longer relative telomere length, which is a sign of a reduced stress level (Sirovnik et al. 2025). However, it must be kept in mind that nose-flaps can cause lesions on the calf’s nose (Kirk and Tucker 2023). In summary, despite some stress associated reactions to calf separation, MMY increases during weaning. Probably, the positive effect of reduced milk intake by the calf exceeds negative effects of weaning distress on milkability.

### Milk flow profiles

The efficiency of milking can be assessed by means of milk flow rates, durations of the different milking phases and the appearance of milk flow curves (Sagi et al. 1980; Yadav and Sharma 1985). In nursing cows, decreased average milk flow rates at milking were found in several studies (Mendoza et al. 2010; Zipp et al. 2018; Rell et al. 2024). Bimodal milk flow curves indicate an interruption or transient reduction of the milk flow between the cisternal and the alveolar fractions (the cistern is emptied before milk is released from the alveolar compartment; Bruckmaier and Blum 1998; Dodenhoff et al. 1999). To identify bimodal milk flow, the Lactocorder (WMB AG, Balgach, Switzerland) has been frequently used in recent decades. It is a portable or stationary milk meter that asesses the milk flow continuously. Another promising device is the VaDia vacuum recorder (Biocontrol, Rakkestad, Norway) which showed moderate agreement (κ = 0.59) with bimodality detected via Lactodorder, with high specificity (0.92) (Wieland et al. 2021). It should be kept in mind that bimodality is not always clear-cut as the milk flow does not always drop to zero. Therefore, classification of different levels of bimodality as used by (Tuor et al. 2022) may provide a better insight. Furthermore, most AMS units can assess milk flow on quarter level, which gives more specific information. Vacuum and pulsation can be automatically adjusted at each teat cup according to milk flow (reviewed by Upton et al. 2025).

In DCC systems, due to the calves’ suckling activity, udder fill may be low at the beginning of milking and may therefore cause bimodal milk flow curves (Rell et al. 2024). On the other hand, if all cisternal milk is suckled by the calf before milking (which is possible in some DCC systems), no bimodal milk flow curves will be visible, but overmilking can occur at the start of the milking process. In previously suckled quarters, both bimodal milk flow curves and overmilking are more likely to occur than in evenly filled udder quarters. Conversely, if restricted nursing occurs directly before milking, the calf may not empty the udder completely, and ejected milk would continue flowing into the cistern. In this scenario, if all alveolar milk was already shifted to the cistern, no bimodality would be visible either. An increased incidence of bimodal milk flow curves was found by Barth et al. (2007), while Rell et al. (2024) found no difference. It must be considered that Rell et al. (2024) used cows from the same study farms but at later lactational stages as controls (non-nursing cows: 194.3 days in milk versus nursing cows: 54.2 days in milk). Cows in late lactation are also more susceptible to bimodal milk flow profiles (Bruckmaier and Hilger 2001). Barth et al. (2007) used control cows of the same lactational stage as dams. In Rell et al. (2024), on the CAM farms, 44% of the milking curves of non-nursing cows were bimodal, while 28% of the milking curves of nursing cows were bimodal. This corresponds well with the occurrence of bimodal milk flow curves in the CAM group in Barth et al. (2007: 25% of all milkings). As the required prestimulation time also increases in the course of lactation (Bruckmaier and Hilger 2001), the effect of late lactation might have exceeded the effect of nursing and prevented the detection of statistical significance in Rell et al. (2024). Nevertheless, in the context of available literature, rates of 25% or 28% do not reflect an increased incidence of bimodality. Other researchers, using a Lactocorder, reported 35.1% (Sandrucci et al. 2007), 33.8% (Samoré, 2011) and 22% (Dodenhoff et al. 1999) of bimodal milk flow in non-nursing cows (cited by Fernandes et al. 2023). More research is needed to clarify whether the occurrence of bimodal milk flow curves is actually increased in DCC systems.

### Degree of udder evacuation

Incomplete milk removal, has been investigated in several studies by measuring how effectively the udder is emptied. One approach is injecting supraphysiological doses of OT after cluster detachment to extract the unharvested milk. It must be kept in mind that this method also extracts residual milk–which is not removed during normal milking–causing an overestimation of the amount of removable milk. Residual milk represents between 3% (Ferneborg et al. 2016; assessed in AMS on a quarter level) and 20% in addition to the available milk (Jenni et al. 2024; in a milking parlor), meaning that the actual milk loss cannot be evaluated with this method. Nevertheless, using this method, De Passillé et al. (2008) found 5.5 kg (36.8%) more unharvested milk in nursing dams in a CAM system compared to non-nursing animals. This is consistent with results of Ferneborg et al. (2024), who found 24% unharvested milk during WDC, 18% during subsequent part-time contact and 11% two weeks after separation. Incomplete udder evacuation after milking in nursing cows may be caused by disturbances of the milk ejection reflex linked to calf suckling. In addition, frequent but short suckling events leaving the udder incompletely evacuated may lead to reduced milk synthesis mediated by autocrine feedback regulation through a glycoprotein (Feedback Inhibitor of Lactation; FIL). Though FIL plays a role in suppressing milk synthesis when milk accumulates in the alveoli, other local factors (eg serotonin, or insulin-like growth factors 1 and 2 and casein-derived peptides) collectively regulate cell turnover within the mammary epithelium. This demonstrates the mammary gland’s capacity for self-regulation of milk production in response to demand (reviewed by Lollivier et al. 2002; Weaver and Hernandez 2016). These mechanisms are presumed to underlie the lower milk yields observed even after weaning, particularly in WDC (see 6.1, Table 2). On the other hand, in the context of DCC rearing, it can be argued that as long as frequent–though partial–udder emptying in combination with milking removes the same daily amount of milk from the mammary gland as compared to a control situation without suckling, milk synthesis should not be locally downregulated.

### Milk composition

Milk fat content increases during the course of milking: Fat globules accumulate within the lactocytes along the apical cell membrane, and their final exocytosis into the alveolar lumen is enforced by the contraction of the myoepithelium (discussed by Hurtaud et al. 2020; found in mice: Masedunskas et al. 2017). In addition, capillary forces appear to retain fat droplets in the alveoli and small ducts of the alveolar tissue. This results in a continuously increasing fat percentage in the removed milk over the course of a milking (Ontsouka et al. 2003). This allows conclusions on the level of udder evacuation and also to identify a failure of OT release as the underlying cause based on milk samples. Nevertheless, fat content must be seen in light of between-cow, between-day, between-milking and between-quarter variation. For example, day-to-day variation can be up to 8% and milking-to-milking variation can be up to 5.7% (Forsbäck et al. 2010). Therefore, adequate study designs should be chosen to minimize these effects.

Reduced fat levels of 0.5–1.5% have been found in samples of whole-milking samples in nursing compared to non-nursing dams in numerous studies independently from the daily contact management (Boden and Leaver 1994; Bar-Peled et al. 1995; Mendoza et al. 2010; Zipp et al. 2018; Barth 2020; Nicolao et al. 2022; Wenker et al. 2022b; Ospina Rios et al. 2023; McPherson et al. 2024). Increased frequency of udder evacuation by milking (in non-nursing cows) reduces the fat content per milking but increases or does not affect the fat yield per day (reviewed by: Sanchez-Duarte et al. 2020). In the study by Bar-Peled et al. (1995), 6 × milking or 3 × milking and 3 × restrictive suckling was compared to 3 × milking without suckling. Fat contents of the harvested milk were not significantly different between the groups, except that 3 × suckling and 3 × milking led to lower fat contents than 3 × milking alone. Fat yield was highest in the 6 × milking group, while in the 3 × milking and the 3 × suckling group it was intermediate but still higher than in the 3 × milking group. This indicates that replacing additional milkings with suckling events can intensify the reducing effect of frequent milking on fat content and that possible benefits of frequent milking for daily fat yield are reduced. In most studies with nursing cows (including in this one), reduced fat contents are combined with reduced MMY in suckled dams pointing to suboptimal milk-letdown, and fat-rich fractions remaining in the udder. Interestingly, even when MMY was comparable to non-nursing cows during morning milking, Zipp and Knierim (2024) found reduced milk fat in dams with daytime contact after they spent the night without the calf. In this case, incomplete milk ejection was not caused by low udder fill before milking, so other factors (discussed further in chapters 8.3. and 9.1.) must have been the reason.

Also, reduced fat contents have been found in strip milk samples in different DCC systems (Barth 2024; Rell et al. 2024). At the same time, these studies showed high variations among DCC cows, which is discussed in chapter 7.1. No carryover effects of nursing on milk fat content after weaning were found (Ospina Rios et al. 2023; Bryant et al. 2024; Johanssen et al. 2024; McPherson et al. 2024; Sørby et al. 2024). However, as milk pricing can depend on the composition of the bulk tank milk, reduced fat contents could be economically challenging for farmers (particularly when calving is organized seasonally).

Current study results regarding the effect of nursing on milk protein levels are inconsistent and cannot be related to specific DCC systems: Some authors found elevated levels of protein in nursing cows (Boden and Leaver 1994; Schneider et al. 2007; Barth 2020; Nicolao et al. 2022; Ospina Rios et al. 2023; Zipp and Knierim 2024). In other studies protein levels were similar between nursing and non-nursing cows (Bar-Peled et al. 1995; Margerison et al. 2002; Mendoza et al. 2010; Nicolao et al. 2022; Wenker et al. 2022b; Johanssen et al. 2024; McPherson et al. 2024; Zipp and Knierim 2024), while Cozma et al. (2013) found reduced protein levels in suckled cows. In addiction to milking frequency, the protein content is influenced by a variety of factors including parity (Morales Piñeyrúa et al. 2018), breed (Chen et al. 2017) and nutrition (Hullár and Brand 1993; Chen et al. 2017). As the milk protein content has economic effects for farmers and the type of protein influences the properties of the milk during cheese production (Soodam and Guinee 2018), deeper investigation of the link between nursing and milk protein contents and composition are of interest.

Lactose was found to be elevated (Boden and Leaver 1994), unaffected (Fulkerson et al. 1978; Sandoval-Castro et al. 1999; Lupoli et al. 2001; McPherson et al. 2024) or reduced (Barth et al. 2007; Zipp et al. 2018; Johanssen et al. 2024; McPherson et al. 2024) in nursing cows. In non-nursing dairy cows incomplete udder emptying (Penry et al. 2017) or once-daily milking is also associated with reduced milk lactose concentrations (eg Stelwagen et al. 1998; Lacy-Hulbert et al. 1999; Delamaire and Guinard-Flament 2006). Tight junctions, which maintain the polarized potential between the blood and milk sides of the mammary epithelial cells and thereby prevent the exchange of ions and small molecules between blood and milk (reviewed by Stelwagen and Singh 2014; Cai et al. 2018), are thought to play a role in this process. When milk remains in the udder, tight junctions become leaky after 18–21 h allowing lactose to diffuse from milk into the blood plasma (reviewed by Stelwagen and Singh 2014). If lactose content in the milk of alveoli is reduced, the osmotic pressure decreases and less water is transferred from blood into the milk and therefore milk yield decreases (reviewed by Cai et al. 2018). Current study results do not allow conclusions on effects of nursing on milk lactose, nor on underlying reasons.

An increased level of milk protein and lactose in bulk milk in combination with problems of complete udder evacuation during DCC is an interesting phenomenon, as the glycoprotein FIL, which downregulates milk synthesis, if the udder is not emptied regularly, also inhibits the synthesis of casein and lactose (reviewed by Lollivier et al. 2002).

### Udder health

According to current literature, somatic cell counts in harvested milk are unaffected (Krohn 2001; Barth et al. 2007; Zipp et al. 2018; Barth 2020; Nicolao et al. 2022; Wenker et al. 2022b; Zipp and Knierim 2024) or reduced (Cozma et al. 2013) in nursing cows compared to non-nursing cows. This is consistent with Beaver et al. (2019), who found beneficial effects of suckling on udder health and mastitis risk in their systematic review (based mainly on studies with restricted DCC). González-Sedano et al. (2010) found that short-time CAM (referred to as residual suckling) reduces the risk of clinical mastitis in Holstein X Zebu cows. Studies with longer follow-up periods, larger sample sizes and inclusion of cows with (sub-)clinical mastitis would be helpful to confirm possible positive effects of nursing. Effective, complete and frequent udder quarter evacuation (Fröberg et al. 2007) together with the bactericidal effect of lysozyme (Ellison and Giehl 1991) from calves’ saliva are possible explanations for the potentially protective effect of suckling on udder health. Nevertheless, complete evacuation of udder quarters is probably more related to older calves as suckling bouts become fewer and longer with increasing calf age. Little research exists on how suckling affects teat condition in dairy cows. It can be assumed that stress on the teats is higher in systems where multiple calves suckle one udder. In studies where udders were suckled by one calf, suckling caused rougher teat skin than milking but lowered the amount of esculin-positive bacteria on the teat (Rasmussen and Larsen 1998) and caused no differences in teat end hyperkeratosis (McPherson et al. 2024), teat wall thickness (Dahlberg et al. 2021) or the occurrence of swellings at the teat base at milking (Rell et al. 2024).

Hamann and Stanitzke (1990) demonstrated increased teat diameters (1.2–2.7 mm) after milking compared to suckling (0.2 mm). Milking increased teat end thickness by 7.9% more than suckling. Teat end temperature increased with calf suckling but decreased with milking, suggesting a higher risk of infection after milking than after nursing. Those results support the conclusion that continuous vacuum and cyclic liner compression cause more strain on the teats than calf suckling (see chapter 4.3.). It should be borne in mind that these findings of similarly good or better udder health were achieved in DCC without teat disinfection after milking, while in some studies control cows were dipped (Barth 2020; Zipp and Knierim 2024). When cows have calf contact directly after milking, teats should not be dipped with a disinfectant solution (Barth et al. 2022). For teat preparation before milking usually no disinfectants or chemical cleaning products are used (Rell et al. 2024; data not shown).

It can be concluded that DCC rearing entails reduced MMY during the suckling period, with possible carryover effects after weaning. Practical implications of reduced fat content in bulk tank milk depend on various factors such as the proportion of the herd nursing a calf at a given time, the contact duration or the seasonality of calvings. Further possible effects of DCC rearing compared to early separation related to milking include improved or unaffected udder health and teat condition, incomplete udder evacuation and alterations in protein and lactose contents. Consdering the different types of daily DCC (WDC, daytime contact, nighttime contact or restricted CBM or CAM) and their relevance to milkability, the following aspects should be considered: MMY is reduced during the suckling period in all contact systems except highly restricted CAM (eg Cozma et al. 2013). Studies where different contact types were compared show that MMY is not always lower if the contact time is longer (McPherson et al. 2024; Rell et al. 2024), but as shown in Table 2, overall, relative MMY reductions were highest in WDC and CBM, intermediate in HDC and lowest in CAM systems (means of RC from Table 2: CBM: 55.6%, WDC: 52.4%, nighttime contact: 45.6%, daytime contact: 39.5%, CAM: 25.7%). Average milk flow, an important parameter determining milking time, correlates strongly with MMY (Rell et al. 2024), and should therefore increase at higher levels of udder fill when the full amount of available milk can be extracted without milk ejection disorder. Fat content reductions were mostly found to be independent of the contact type (Barth 2020; McPherson et al. 2024; Rell et al. 2024; Zipp and Knierim 2024).

## Milk ejection disorders in DCC rearing

### Identification of milk ejection disorders

Most of the parameters measured in the previously cited studies (MMY, milk flow, bimodality, milk fat content) indirectly inform about milkability in a non-invasive way. However, alterations of these parameters–except for milk fat content–may result not only from actual milk ejection disorders but also from other factors that reduce the milk flow through the teats, the amount of milk synthesized and secreted into the alveoli or delayed milk ejection (ie due to inappropriate milking machine settings and milking routines). In a study on non-nursing cows by Belo et al. (2009), 31% of cows classified by farmers as “affected by milk ejection disorder” actually had anatomical disorders or no disorder at all. This demonstrates that without technical equipment, localization of the source of milkability problems may be inadequate (Belo et al. 2009).

A true dysfunction of the milk ejection reflex is caused by insufficient OT release from the pituitary gland. Unambiguous identification therefore requires measurements of plasma OT concentrations or intramammary pressure (Bruckmaier et al. 1996). Both apporaches are invasive and are nowadays rarely used for animal welfare reasons. In CCC systems, the risk of injury to cannulated cows may be further increased due to cow–calf interactions. Determining the amount of unharvested milk after cessation of spontaneous milk flow as described in chapter 6.3. is less invasive but still not suitable under practical farm conditions. The assessment of OT in bovine saliva has been validated by Lürzel et al. (2020) and used in DCC, but not in the context of milk ejection by Neave et al. (2024b). Recently, Wellnitz et al. (2025) demonstrated that salivary OT concentrations remain unchanged during 20 min after i.v. injection of different doses (0.5, 1, 5, and 10 IU). This demonstrates that salivary OT levels are not suitable for capturing the short-term dynamics of OT concentrations needed to identify milk ejection disorders. A feasible on-farm method is the analysis of fat content in strip milk samples collected after cluster detachment as alveolar fat secretion depends on OT-induced myoepithelial contraction (Masedunskas et al. 2017; see chapter 6.4. for a detailed explanation).

**Figure 1:**
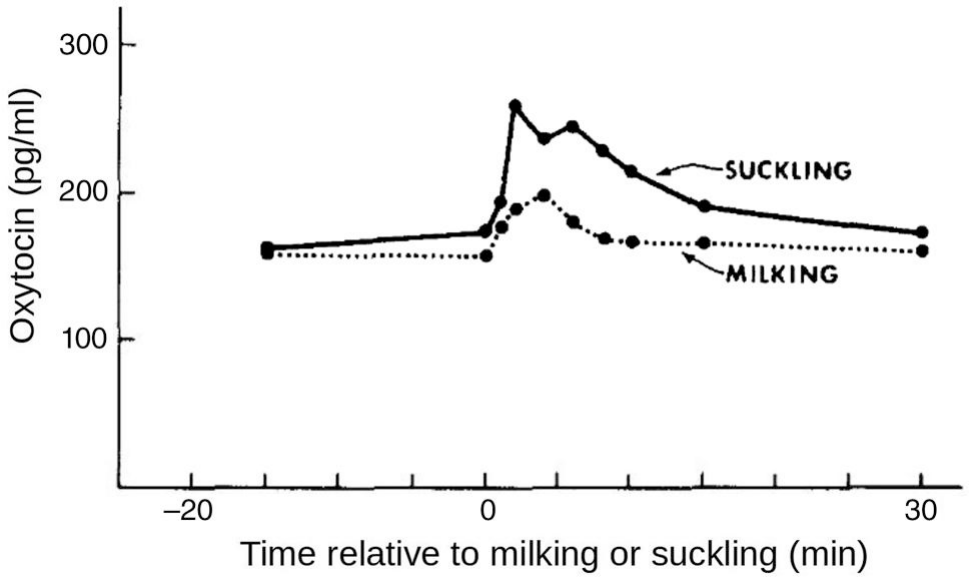
Mean serum oxytocin concentrations measured in response to milking or suckling in cows housed with their calves (Akers and Lefcourt 1982).

Recently it has been shown under experimental conditions, that in non-nursing dairy cows, milk fat content does not further increase further once the OT action is stopped preterm (Jenni et al. 2024). In the study by Jenni et al. (2024) an OT-receptor-blocking agent (Atosiban) was used to simulate the sudden cessation of OT action. This resulted in a consistently low milk fat contents at all levels of incomplete milk ejection (mean milk fat concentration at a spontaneous udder emptying of < 20%, 20–40% or 40–60%: 2.82–2.96 g/100g). Only when spontaneous udder emptying exceeded 80% did milk fat content increase substantially to 6.46 g/100g (there was an unexpected data lack from 60–80% of spontaneous udder emptying). Most likely, OT-receptor blockade caused a sudden cessation of myoepithalial contraction and stop of fat secretion although milk flow kept going for a short time. Thus, OT receptor blockade effectively mimics the stop of OT action. This may therefore be a more adequate parameter (compared to fat contents at ongoing OT action which is limited to experimental settings) to estimate changes in milk fat content when the milk ejection reflex is disturbed. As an on-farm method, strip milk fat content is a suitable parameter to distinguish between complete and incomplete udder emptying due to milk ejection disorder. It may be combined with measurements of electric conductivity (Jenni et al. 2024). Electric conductivity is affected by various other factors such as milk’s ionic balance (indicating udder infection), breed, parity, lactation stage or milking interval which reduces its explanatory value (cited from: Zaninelli and Tangorra 2007). Reduced strip milk fat contents in nursing compared to non-nursing cows were found by Barth (2024; WDC) and Rell et al. (2024; different contact types). Strip milk fat contents, especially of the nursing cows showed high variability. In Barth (2024), values ranged from 2.0 to 26.0 g/100 g (median: 7.0–8.0 g/100 g; Fig. 1), and in Rell et al. (2024) from 2.5 to 17.0 g/100 g (median: 6.0–9.0 g/100 g). The range of fat content in strip milk samples of non-nursing cows in the same studies was narrower (Barth 2024: 2.5–15.0 g/100 g, median: 9.0–11.0 g/100 g (from Fig. 1); Rell et al. 2024: 3.0–12.5 g/100 g, median: 7.0–8.5 g/100 g). Fat contents should therefore always be interpreted within the context of the corresponding herd to allow consideration of factors such as feeding, breed, stage of lactation, milking procedure and measurement technique. In addition to individual cow susceptibility to disturbed milk ejection, the degree of udder fill at the start of milking may also influence strip milk fat content. The data of Rell et al. (2024), showed no significant difference between the DCC systems, but numerically, the median of CBM cows’ strip milk fat content was higher than that of all other non-nursing control groups. One reason for this effect of CBM might be that the low-fat (cisternal) milk fraction was already removed by calf suckling before milking (as discussed by Rell et al. 2024). Jenni et al. (2024) found increased strip milk fat contents when udders were emptied to more than 80%. Therefore, high fat content in strip milk samples could either mean, that the dam experienced complete milk ejection during milking or–in cases like mixed dam-foster systems or low-yielding breeds–calves had nearly emptied the udder before milking. The last-milked milk (ie originally the alveolar fraction), high in fat, ejected during suckling could still be harvested from the cistern during milking, independent of milk ejection at that time. Therefore, a low fat content in strip milk or whole milk samples is a sign of impaired milk ejection, but a high fat content in strip milk should rather be interpreted as a sign of a relatively empty udder before milking, at least in CBM systems. While the individual fat content of the whole milking was analyzed in several studies independent from DCC system (eg Barth 2020; Zipp et al. 2018; Nicolao et al. 2022; Zipp and Knierim 2024), fat content of strip milk has so far rarely been used (Barth 2024; Rell et al. 2024). The influences of DCC system and the lag time between suckling and milking, which can vary within systems, on milk ejection and udder emptiness after milking should be further investigated using strip milk samples as an indicator of udder emptiness.

### Effects of suckling versus milking on OT release

In the past, several studies were performed to describe the increase in plasma OT levels in response to suckling or milking. As this method requires the placement of permanent venous cannulas, these studies were typically performed in a controlled experimental setting, with low numbers of animals. Most studies focused on the first days postpartum. Several older studies found that suckling elicited significantly greater increases in OT concentrations than milking in the nursing cows (Akers and Lefcourt 1982; Akers and Lefcourt 1984; Lupoli et al. 2001; Tancin et al. 2001; De Passillé et al. 2008). There were no significant differences in OT levels between non-nursing cows at milking and nursing cows at nursing (Tancin et al. 2001; De Passillé et al. 2008), indicating an inhibition of OT release at milking rather than an augmentation due to calf suckling in nursing cows. The cited studies therefore provide evidence that, in nursing cows, suckling is a more effective stimulus for plasma OT increase than milking. Given that primiparous non-nursing cows in the post partum phase appear to be highly susceptible to milk ejection disorders, it should be noted that parity was not considered in the previously cited studies (which used either only primiparous or only multiparous cows). Only Tancin et al. (2001) found higher OT levels in suckled primiparous than in suckled multiparous cows in response to milking in the post partum phase which may suggest that calf contact has protective effects regarding milk ejection disorders in the post partum phase.

Individual variations in OT increases between cows are high, even in non-nursing cows (Mayer et al. 1991) and it must be considered that reduced OT levels do not necessarily impede milk ejection, as long as the cow-individual threshold level is exceeded during the whole milking process.

Direct comparison of OT concentrations measured in the previously named studies must be made with caution as standardization of assay techniques for OT concentrations is not ensured–differences in sample preparation, assay performance, and laboratory-associated factors all affect measured values (MacLean et al. 2019). Schams et al. (1984) showed that OT release can vary greatly from milking to milking, while the milk-flow curve remains almost identical. Bruckmaier et al. (1994) demonstrated that with repeated OT injections–even at different doses–the same maximum intramammary pressure was always reached. With a continuous OT infusion leading to a steady increase in plama OT, the intramammary pressure rose to a plateau shortly after the start of the infusion and remained there until the end. In cases of impaired milk ejection, there can be a grey area, i.e., partial ejections, which are primarily due to OT release ending prematurely.

It could be suspected that frequent increases of plasma OT due to multiple suckling events per day might lead to elevated threshold levels and thereby cause milk-ejection problems at milking. Injection of OT causes desensitization of the mammary gland, leading to poor responses to natural OT release (Macuhová et al. 2002; Belo and Bruckmaier 2010). Although OT receptor desensitization due to more frequent OT release induced by calf suckling has not yet been demonstrated, it is physiologically conceivable.

## Factors affecting milk ejection in nursing dams

### Cow-Level: Dam-individual variation

Despite an overall decrease in MMY, variation among nursing cows is high (eg Barth 2020; Johnsen et al. 2021a; Mutua and Haskell 2022; Sørby et al. 2023; Ferneborg et al. 2024; Hanssen et al. 2024; Neave et al. 2024a), indicating that individual factors (also discussed in chapter 5.2.3.) play an important role in the ability to release milk at milking during the nursing phase.

Farmer experience supports the idea that the susceptibility of nursing cows to milk ejection disorders is connected to cow-individual characteristics (Rell et al. 2024). In the experiment by Tancin et al. (2001), not all nursing dams were affected by OT inhibition in response to milking, but individual cows released no OT at all in response to milking. In the same study, suckling was reintroduced after four weeks of cow-calf separation (only milking) to test OT responses to the first suckling after conditioning to milking. Reactions were highly variable reaching from no OT release in response to the first suckling to no OT release in response to the subsequent milking (Tancin et al. 2001).

As stable animal characteristics mediate behavioral responses to milking, this could be utilized in genetic selection (Van Reenen et al. 2002) to improve milk let-down in nursing dams. However, the importance of good maternity traits must be kept in mind. Ospina Rios et al. (2023) found that “bold” cows showed less maternal behavior than “anxious” or “sociable” cows in a half-day DCC system, but MMY was not assessed. The quality of maternal abilities may be connected to milkability in response to machine milking. Selecting for dams that easily release milk in the milking parlor–as it has been done in high-yielding dairy breeds for decades (Miglior et al. 2017)—might therefore suppress other essential traits such as postpartum calf care or initiation of nursing bouts by the dam during the first days postpartum. In low-yielding breeds without additional calf feeding, it remains unclear if milk production is sufficient to adequately nourish a calf while also releasing acceptable amounts of milk in the milking parlour. Overall, individual cow characteristics related to maternal abilities and their consequences for machine milking only become visible in DCC systems, thereby adding variability to milkability parameters.

### Udder level: Low degree of udder fill at the start of milking

Overall, on udder level, short intervals between udder evacuations–causing low udder fills at the start of milking–appear to be the most obvious reason for delayed or poor milk let-down at milking in nursing cows. The duration between suckling and milking and the number of calves suckling the cow (the cow’s own calf only or mixed dam-foster-cow contact with up to four calves per cow) influence quarter filling at milking. In CBM, CAM and HDC, the lag time between suckling and milking at the milking time after the separation phase, can be estimated. In WDC, and the milking following the contact phase in HDC, this is not the case, unless suckling is observed or assessed by sensors. Even within the CBM system there is high variance between farms and studies concerning the daily contact duration (eg Ivemeyer et al. 2016: 45–60 min; Barth 2020: 15 min., Ivemeyer et al. 2021: time between suckling and milking on six commercial farms with CBM or CAM: 0.4–3 h. mean = 1 h; Nicolao et al. 2022: 10 or 20 min). Assuming that suckling occurs at the start of the contact phase, the lag time between nursing and milking equals the contact duration minus nursing duration, plus any waiting time in front of the milking facility after contact. The specific lag time was so far not assessed systematically. If the lag time between suckling and start of pre-stripping is less than two minutes, it is possible that the milk ejection from suckling can be maintained until milking by machine begins. However, this is theoretical, based on the half-life of OT, and possible stress reactions that may be experienced by the cow can impede continuous above-threshold OT concentrations. If the lag time between suckling and milking exceeds two minutes, a new milk ejection will occur provided there is no central inhibition of OT (Bruckmaier 2001). In non-nursing cows, when milking intervals are shorter than 4 h, no cisternal milk is available for extraction even before the milk ejection reflex is elicited (Knight et al. 1994). When only the cow’s own calf is suckling, milk will not be removed completely from the udder after nursing, but individual quarter fill will differ at the start of milking. Smart gates have already been used to restrict DCC to periods starting 5.5 h after milking until a successful AMS milking was recorded, tested with four cow-calf pairs (Johnsen et al. 2021a). This resulted in higher milk yield compared to free DCC, but also in lower average daily weight gain in these calves (Johnsen et al. 2021a), more allosuckling, and more unrewarded attempts by cows to access their calves (Johnsen et al. 2021b). This method was therefore not recommended from an animal welfare point of view (Johnsen et al. 2021a). Knowledge of the influence of lag time between suckling and milking remains limited, and therefore further investigation is needed.

In contrast to the more complex aspects related to the cow-calf bond or cow-individual factors discussed in the following chapter, udder fill does not affect the amount of OT released from the pituitary gland in response to teat stimulation, but does affect the lag time before milk ejection (Bruckmaier and Hilger 2001). Independently of udder fill and stimulation intensity, Weiss and Bruckmaier (2005) found an increase in plasma OT 30 sec after stimulation began. The lag time between the start of udder stimulation and milk ejection, however, varied between 30 and 150 sec, with a negative correlation between lag time and udder fill of r = 0.54 (Weiss and Bruckmaier 2005). The reason is, that the alveolar surface area remains similar regardless of alveolar fill (Bruckmaier and Hilger 2001). If the alveoli are poorly filled with milk, greater myoepithelial contraction is necessary to transfer milk from the alveoli to the cistern. More time is needed to cause greater contraction of the myoepithelial cells and to move small amounts of alveolar milk through the duct system to the cistern (Bruckmaier and Hilger 2001). In cows with an udder fill of 20–60%, Bruckmaier and Hilger (2001) measured a duration of 80–90 sec from the start of teat stimulation until intramammary pressure increased. Kaskous and Bruckmaier (2011) found that a prestimulation time of 45 sec followed by a latency time of 45 to 60 sec resulted in the highest average milk flow during milking in cows with an udder fill of 20–40%. This total of 90–105 sec from the beginning of prestimulation to cluster attachment is high compared to the findings of Weiss and Bruckmaier (2005), who found no further increase in average milk flow after 60 sec of vibration stimulation without latency time in cows with udder fills of 20–40% and 40–60%. Studies on the influence of prestimulation or latency time before milking on dams milkability are scarce. Neave et al. (2024a) found no influence of preparation duration (mean ± SD: 1.3 ± 1.0 min, range: 0.3–4.3 min) on milk yield of dams. It should also be noted that in the studies mentioned above, MMY did not differ between cows with different udder fills (Weiss and Bruckmaier 2005; Kaskous and Bruckmaier 2011). Zipp et al. (2018) compared pre-stripping and udder cleaning (approximately 20 sec) followed by 40 sec of vibration or manual stimulation in WDC dams and found an increasing effect of manual stimulation on average milk flow, but neither MMY nor fat content differed between treatments. From these findings, an increased need of udder prestimulation and latency time can be postulated in dams with CBM. However, a latency time between end of prestimulation and cluster attachment of 120 sec or longer is not recommended, as it may lead to OT clearance and reduced alveolar contraction (Masedunskas et al. 2017; Bruckmaier et al. 1994).

Even though, milk yield did not increase with longer prestimulation and latency time in the studies mentioned above, optimal stimulation and latency time are important to prevent overmilking at the start of milking (reviewed by Upton et al. 2025) and the associated bimodal milk flow curves when milk was present in the cistern before milking began. Due to different quarter fills and possibly empty quarters during milking, the risk of overmilking in dams with CBM is high. Results are conflicting as to whether overmilking leads to teat end hyperkeratosis, which can be associated with a higher risk of mastitis (Cerqueira et al. 2018), or not (discussed by Upton et al. 2025). However, it is clear that teat tissue can be stressed by high vacuum during low or stopped milk flow (reviewed by Upton et al. 2025) and the finding that overmilked cows showed more stepping behavior can be interpreted as a sign of discomfort (Cerqueira et al. 2017). Beyond animal welfare concerns, overmilking reduces milking efficiency (Upton et al. 2025). To prevent bimodal milk flow or overmilking after cluster attachment, increased prestimulation and latency time are necessary. The efficiency of stimulation by hand, vibration, high pulsation frequency and low vacuum–whether in milking parlors or in AMS–for inducing OT release is comparable in non-nursing cows (reviewed by Upton et al. 2025), and prestimulation and latency time can be increased across all systems at udder level (vibration stimulation: eg Watters et al. 2015: time-based; Singh et al. 2024: milk flow-based). However, only quarter-individual milking with milk flow dependent vacuum and pulsation (Reinemann et al. 2025)—most common in AMS (Upton et al. 2025)—can meet the needs of cows with differing quarter fill.

If no AMS is used, quarter-individual teat-cup removal can be performed manually. Transparent milk tubes are essential for observing quarter-specific milk flow; empty quarters are detached and closed with a dummy plug (Zipp and Knierim 2024). However, this is time-consuming, and detection of empty quarters depends on the milker’s ability. Manual teat-cup removal also causes a vacuum drop and respray, potentially introducing pathogens into the teat canal, and milking with one or more cups detached can misalign the cluster and increase load on the remaining quarters, stressing teat tissue. Automatic cluster removal based on aggregate milk flow does not reliably prevent quarter-level overmilking, even in non-nursing cows. However, the occurrence of overmilking depended on the cluster removal milk flow threshold (Krawczel et al. 2017; Fernandes et al. 2023).

Adapting the prestimulation and latency time and controlling the start and stop of milk removal based on quarter-milk flow–as it is already possible in AMS–may help to improve milk flow profiles in dams. Several DCC studies have been conducted with AMS milking. Unfortunately, no details were provided about the prestimulation time or method, or whether milking start and stop were quarter-individual (Johnsen et al. 2021a; Churakov et al. 2023; Hanssen et al. 2024; Johanssen et al. 2024; Sørby et al. 2024). Future studies should report details about milking facility parameters and dam milk flow data to allow better interpretation of results. The finding that low milk fat content was observed alongside high MMY in HDC dams during morning milking after overnight separation (Zipp and Knierim 2024) indicates that udder fill before milking is not the only factor influencing milk ejection in dams: individual (8.1), neuroendocrine, and behavior-related factors are discussed in the following chapters.

### Central level: stress at milking

As previously discussed stress affects milkability independently of nursing (see chapter 5.2.). However, in DCC rearing, the separation of cow and calf during milking adds a potent stressor. Schneider et al. (2007) observed more vocalization and tense behavior during milking in CAM cows than in control animals, while WDC dams showed intermediate responses (score combined: eye wideness, tense head position and absence of rumination). Zipp et al. (2018) also reported more milkings with a tense head position and, additionally, more defecation in WDC dams compared to non-nursing cows. Roadknight et al. (2022) found increased stepping and kicking during the milking in dams with nighttime contact before reunion with their calves. However, in other or the same studies as mentioned above, no differences were found between dams and control cows regarding stress associated behavior during milking (Schneider et al. 2007: elimination; kicking; Zipp et al. 2018: rumination, stepping, kicking; Roadknight et al. 2022: vocalization during separation, rumination; Neave et al. 2024a: stepping and kicking; Rell et al. 2024: hind leg activity). Schneider et al. (2007) further observed that vocaliziation and tense behavior during milking decreased during the course of lactation (data collected during the first three months of lactation). Calves were nursed for three months, and elevated behavioral respones during milking persisted for approximately 55 days in WDC and 75 days in CAM dams. Milk cortisol in dams, used as an indicator of stress, did not differ from controls (Roadknight et al. 2022). Although some behavioral signs of stress were observed, Zipp et al. (2018) even detected activation of the vagal axis based on heart rate variability measurements. The authors suggested that milking might represent a mildly stressful event for WDC dams, but that dams may generally be more relaxed than non-nursing cows due to more frequent OT release throughout the day by nursing and milking (Akers and Lefcourt 1982; Tancin et al. 2001; De Passillé et al. 2008), as OT activates the vagal axis (reviewed by Uvnäs-Moberg and Petersson 2005). Overall, nursing cows in different DCC-systems shown some behavioral signs of stress during milking, while physiological parameters remained unaffected. However, it cannot be excluded that some behavioral reactions in nursing dams are attributable to discomfort caused by overmilking of individual quarters rather than to stress due to calf separation. In HDC systems after the separation phase, and CAM systems, anticipation of reunion with the calf may also influence behavior during milking. Further research focusing on physiological parameters are needed to refine the interpretation of behavioral findings. Neave et al. (2024a) reported high individual variation in restless behavior (ie stepping and kicking) in the milking parlor, independent of treatment, and found that stepping and kicking were negatively associated with MMY. It remains unclear whether this is a true correlation between MMY and restless behavior, as both could reflect stress. Alternatively, there may be a third factor affecting both variables. For example, a higher fly burden in the evening milking could increase kicking behavior, while a shorter milking interval compared to the morning milking could result in lower MMY. When nursing and non-nursing dams were exposed to an unfamiliar environment (milking parlor), inhibition of OT release in response to milking was more pronounced in non-nursing primiparous cows than in suckled cows. Total inhibition of OT release in response to milking was observed in four out of seven non-nursing cows, but in none of the nursing cows (Tancin et al. 2001). Reduced sensitivity to stress in nursing cows may be mediated by numerically elevated basal OT levels (Bar-Peled et al. 1995; Lupoli et al. 2001; De Passillé et al. 2008) or by more frequent OT release in nursing cows during the day through both nursing and milking. Oxytocin can centrally attenuate activity of the hypothalamo-pituitary-adrenal axis and thereby reduce the response to stress (see chapter 5.2.4.). It may therefore act as a mediator of the hyporesponsiveness of the hypothalamic-pituitary-adrenal axis during lactation (Slattery and Neumann 2008; Ralph and Tilbrook 2016).

Relating stress during milking to the previously discussed cow-individual variations, transient separation from the calf may be sufficiently stressful in dams with strong maternal instincts to inhibit OT release. This effect may be further enhanced in dams with an anxious temperament. In contrast, a dam which generally copes well with stressful situations and has weaker maternal motivation may not experience imparied milk ejection in the same situation. However, selecting for improved milk let-down during milking carries the risk of loosing maternal abilities, as discussed before.

## The role of the cow-calf bond

In addition to stress–which has well-documented inhibitory effects on OT release during milking–the oxytocinergic system is also modulated by a variety of emotional and social cues and exerts diverse neuromodulatory effects across various brain regions in mammals (Grinevich and Neumann 2021). It is therefore reasonable to hypothesize that the establishment of an emotional cow-calf bond may affect OT response milking stimulation. However, direct investigation of central processes in cattle–such as the brain’s response to calf versus machine stimuli, or the dynamics of OT receptor sensitivity and distribution–is currently unfeasible. Notably, intraspecies differences within bovines regarding OT release in response to milking versus suckling are significant (Sandoval-Castro et al. 1999; Kaskous et al. 2006). Therefore, results of thematically related studies in other species, which are contradictive (Zinaman et al. 1992; Febo 2011), should be applied to cattle with caution.

The following sections introduce two reasonable, though not directly data-based, mechanisms related to the cow-calf relationship that may underlie inhibited OT release at milking in nursing cows.

### Preference of calf nursing over milking

The current state of knowledge regarding dam-calf bonding in water buffalos and other ruminants was recently reviewed by Mota-Rojas et al. (2024): Cow-calf bonding is an early social learning process through which dams form a unique relationship including recognition, selective attachment and maternal care provided exclusively to their own newborn. It has been shown that dams prefer their own calves over foster calves with respect to suckling and laying close together. Calves–especially foster calves–attempt to suckle different cows, which serves their survival (Reinhardt 1980; Wieczorreck and Hillmann 2022). In the following, we focus on the cow. Successful bonding requires the dam’s response to different sensory stimuli (olfactory, tactile, auditory and visual). Oxytocin is the crucial hormone mediating selective maternal behavior via oxytocinergic pathways that process multisensory signals and project them to the relevant cerebral regions. The sensitive period for bonding begins immediately after birth and lasts approximately 6 h in water buffalos (Mota-Rojas et al. 2024). When *Bos indicus* cows were separated from their calf directly after birth for 12 h, they no longer bonded with their calf after reunion (Reinhardt 1980, p. 9, *n* = 2). In *Bos taurus*, five minutes of contact postpartum were sufficient to elicit exclusive maternal behavior towards the own calf after 1–12 h of separation, but after 24 hh the own calf was no longer accepted (Hudson and Mullord 1977). A cow bonded to a calf can be identified by the following behaviors (Sirovnik et al. 2020), which have been observed in DCC-systems: she directs maternal behavior–such as grooming and nursing–towards the calf (eg Johnsen et al. 2015; Franz-Wippermann et al. 2022), and she shows signs of distress when separated from the calf, as observed in CBM (Schneider et al. 2007) and daytime contact (Roadknight et al. 2022; Bertelsen and Jensen 2023). After reunion, restlessness ceases (Sirovnik et al. 2020). Overall, the bond of the cow toward the calf appears to be independent of DCC system. Cows not only attempt to avoid being suckled by calves they are not bonded with, by agonistic interactions (Franz-Wippermann et al. 2022; Roadknight et al. 2022; Bertelsen and Jensen 2023), even OT release in response to suckling by an alien young is reduced compared to suckling by the own offspring (Silveira et al. 1993; Hernandez et al. 2002: goats). When suckling is not allowed, the cow-calf bond appears to be weakened (Johnsen et al. 2015; Wenker et al. 2020; Wenker et al. 2022a). Some authors propose that cows bonded with their calf may voluntarily retain milk in their udders at milking to ensure calf nutrition (Boden and Leaver 1994; Bar-Peled et al. 1995). Milk ejection is an involuntary neuroendocrine reflex that is not consciously controllable; however, hormonal processes related to bonding may alter a dam’s emotional state in a way that causes poor response to milking.

### Calf-associated stimuli and conditioning

Dams are familiar with a variety of tactile, visual, olfactory and auditory stimuli coming from the calf (Orihuela et al. 2021), which may serve as a stronger stimulus than milking facility related stimuli.

Routinely milked cows are, however, used to the sounds, sight and smell related to the milking procedure. Possibly, if the milking routine is optimal, cows may positively associate those stimuli with relaxation experienced through OT effects and the release of udder pressure during the milking process. Studies in women indicate that the secretion of OT can be classically conditioned (Skvortsova et al. 2020a, 2020b). In an early study it has been shown that milk ejection induced by nursing a calf can be conditioned in dairy cows by using an artificial visual stimulus (blue disk). However, the study was conducted with only three animals per treatment (Willis and Mein 1983). In the context of milking, tactile teat stimulation is the main trigger for OT release (reviewed by Bruckmaier and Blum 1998), and to our knowledge, so far there is no evidence that other stimuli than the tactile are sufficient to induce OT release. For example, it has been shown that milk leakage, which can be observed in some cows before manual udder preparation (tactile teat stimulation) in the parlor occurs without OT release but is a sign of high udder pressure and a reduced tone of the sphincter muscle (Bruckmaier 1988; Rovai et al. 2007). Nevertheless, suckling evoked the release of OT and milk ejection in beef cows with denervated mammary glands causing an interruption of the neural pathways from the udder to the brain (Williams et al. 1993). These findings emphasize that factors other than the tactile stimulus can affect milk ejection. This is supported by the study of Peeters et al. (1973), who observed milk ejection during dam-calf interaction, without udder contact, in cows with cannulated teats in a tie stall. In a study with women, peaks of OT were observed even before the start of suckling, often in response to the baby’s cry or vision of the baby showing signs of restlessness in expectation of suckling (McNeilly et al. 1983). In contrast, OT release has been found in response to mechanical breast pumping with amplitudes similar to the release caused by infant suckling, but not before the start of mechanical pumping (reviewed by Uvnäs-Moberg et al. 2020). Exposing nursing dams to different isolated calf-associated stimuli while milking (auditory, olfactory and manual stimulation) did not improve milkability parameters (milk flow, MMY, fat content) compared to normal milking (Zipp et al. 2018). For interpretation of these results, it must, however, be considered that played-back calf calls of alien calves, hair of the own calf and prolonged teat massage were used consecutively in subsequent milkings. Overall, we suggest that in some dams, incomplete or no milk let-down at milking can be caused by habituation to the rich set of stimuli associated with their own calf, which may be a more potential trigger for OT release (in combination with the maternal bond) than the milking machine.

Overall, milkability in nursing dams emerges from an interplay of neuroendocrine, emotional-social, and physiological-mechanical mechanisms presented in chapters 8. and 9. Most of these factors are intertangled to different extents and some of them cannot be measured. The dam-calf bond may alter the hormonal-emotional status of the cow on an underlying level, changing the preconditions for how individual variations in milkability or possible stress-reactions due to calf separation during milking finally take effect. The varibles degree of udder fill before milking, though not completely detached from those factors, is a more predictable aspect that might therefore be easier to solve.

Considering this multifactorial nature of milkability in nursing dams is a good starting point to build an understanding of why milk let-down is more variable and sometimes impaired in DCC systems.

## Zoom-out: how does poor milkability affect practical DCC farming?

### Prevalence

To determine the actual prevalence of milk ejection disorders in DCC systems an epidemiological approach would be necessary. However, the current methods to identify dysfunctions of the milk let-down reflex require technical equipment (analysis of plasma OT levels) or OT administration (measuring unharvested milk), which are too complex and invasive for studies with large animal and farm numbers. Analyzing the fat contents of stripping milk samples is a promising method for future studies performed under practical conditions (Jenni et al. 2024). Analysis of salivary OT may be useful to determine long-term OT levels but not in the context of milk ejection (Neave et al. 2024b; Wellnitz et al. 2025).

From social scientific approaches, it is known that poor milk let-down in nursing dams, contributing to the loss of salable milk, is perceived as an unsolved challenge in practical DCC farming (Vaarst et al. 2020; Vaarst and Christiansen 2023). In a multinational survey study on CCC systems including questions about drivers and barriers, milkability problems were not mentioned as a challenge by farmers (Eriksson et al. 2022). It must be considered that in this study, most of the interviewed farmers used foster cows or a combination of foster cows and dams, which substantially reduces milkability problems. In a combined interview and field study, 5 of 17 of Swiss DCC farmers stated that they rarely had milk let-down problems, while 12 regularly experienced them. The estimated share of affected animals per herd varied between < 10% and 80%. These reports suggest that certain cows might be more susceptible to milk ejection disorders than others but no clear pattern of occurrence was recognizable (Rell et al. 2024). In an interview study by Hautzinger et al. (2025) with 12 DCC and 7 foster CCC farmers, three out of the 12 DCC farmers rated poor milkability as a problem. However, 9 of the 19 (DCC and foster CCC) farmers noticed a worsening in milkability (Hautzinger et al. 2025) after introducing a DCC or foster CCC system. Possibly, poor milkability only becomes a problem, when MMY reductions are not compensated for through adequate milk prices (eg through DCC/CCC labels). A very high response rate of 74,4% to questionnaires about poor milkability, sent to farmers milking exclusively non-nursing cows, emphasized that milkability is an issue of interest not limited to DCC systems (Belo et al. 2009).

### Practical solution approaches

From 17 interviewed farmers, 11 had taken measures to address poor milkability (Rell et al. 2024). Some of these methods have also been tested in scientific studies on milkability with animals with or without calf contact. In a study by Belo et al. (2009) 2% of dairy cows with poor milkability were treated with exogenous OT. Johanssen et al. (2024) treated inhibited milk ejection (defined as low MMY at a perceived high udder fill) with OT injections to prevent mastitis after unsuccessful manual udder massage. Injection of OT is effectful on the short term but over time causes desensitization of OT receptors in the mammary glands (Bruckmaier 2003; Belo and Bruckmaier 2010). Blowing air into the vagina, manual stimulation of the vulva and vagina or transrectal massage of the uterus are other methods to induce OT release and milk let-down through stimulation of the genital tract (Bruckmaier et al. 1993; Belo et al. 2009). These methods are used on some practical DCC farms (Rell et al. 2024) although they are labor-intensive.

Furthermore, it has been shown that feeding concentrate before or during milking can cause an enhanced OT release (water buffalos: Borghese et al. 2007; cows: Svennersten et al. 1990; Johansson et al. 1999). This method has, so far, not been systematically tested in dams. In breeds where milk let-down requires calf presence, a common practice is to allow a very short suckling contact to evoke milk let-down and immediately remove the calf to start milking (Cozma et al. 2017). Another option which is recommended under commercial conditions is to let the calf approach the cow during milking without being able to suckle (in addition to the daily contact time where suckling is possible; Barth et al. 2022), or to let the calf suckle one teat while the other teats are milked (Lucht 2009). Both approaches are effectful, but highly labor-intensive. When suckling is only allowed to trigger milk ejection, malnutrition of calves is a likely consequence (Tournadre et al. 2008; Cozma et al. 2013). Organizing the timing of suckling and milking in a way that udders are full at the beginning of one milking per day (HDC) or impeding calf suckling during some hours before each milking (CAM) is often used in practical farming, when calves are older (Rell et al. 2024). As stated before, only very restrictive CAM actually leads to improved milk yields. This may rather reflect an option for older calves that are not dependent on milk intake anymore in the course of weaning. Studies showed, that cows with contact to their own calf, but without the possibility to nurse it (udder net or calf in an adjacent pen), did not have lower MMY (Johnsen et al. 2015; Wenker et al. 2022b) or fat contents (Wenker et al. 2022b) than non-nursing cows. A system impeding suckling completely, while allowing calf contact, however, is not in accordance with natural behavior and it is doubtful that the demand for more naturalness and animal welfare in dairy production would be met by this method. Besides the already mentioned methods, some Swiss DCC farmers try to improve milkability using homeopathic treatments (Rell et al. 2024). There is no scientific evidence for the effectiveness of homeopathics in this context.

Looking at milking technology, quarter-individual milk-flow based pulsation and vacuum represent a promising tool to enable effective milking when quarter filling are unbalanced. Automatic milking systems offer this opportunity. Studies comparing parlor and AMS milking would be helpful to quantify possible advantages of AMS for milkability.

In conclusion, there are different practical approaches to improve milkability, which are often labor-intensive, in some cases not sustainable, or their efficiency is not proven. Farmers may use them for individual, severe cases, but not generally. Milking vacuum and pulsation on the individual quarter level in AMS represents a promising option for the future of DCC rearing.

## Conclusions

Calf intake does not fully account for observed MMY reductions in DCC systems. Milkability parameters can additionally be affected at udder level, as frequent suckling may lead to a low degree of udder fill at the onset of milking, resulting in prolonged lag times before milk flow starts. Nursing cows show reduced OT release in response to milking compared to suckling, leading to disturbances in the milk ejection reflex. Underlying mechanisms remain incompletely understood but are likely influenced by the emotional bond between dam and calf. This bond may modulate the oxytocinergic system, altering responsiveness to milking stimuli and favoring calf-associated cues over those of the milking environment. However, highly resource-intensive neuroscience research would be needed to explore which brain regions are activated, how oxytocin receptor distribution and activity are affected, and what this means for milk ejection at milking in DCC systems. Such investigations are unfeasible in cattle, and it can be debated whether the results would ultimately hold practical relevance.

To improve milkability in nursing dams, feasible measures such as optimizing the timing of suckling relative to milking should be further investigated. Fat content analysis of complete milkings or strip milk samples currently represents the only feasible on-farm method for larger samples to quantify the prevalence of incomplete udder evacuation.

Milk ejection disorders are not limited to nursing cows, but some influencing factors, such as stress at milking or individual responsiveness to milking machine stimuli, take on specific facets in the context of calf contact and nursing. The prevalence of milk ejection disorders in non-nursing dairy cows requires renewed attention in order to estimate the extent of challenges observed in DCC rearing. Finally, the actual financial loss is not determined by MMY reductions, but should be evaluated on farm level, also accounting for cost saving factors as improved udder health, better calf growth or reduced work-time for calf care.

Finally, future research should more deeply investigate the genetic factors that influence milk let-down in nursing dams. While heritability of milkability traits has been reported, little is known about how these parameters behave in the context of dam-calf contact (DCC) systems (eg if good maternal traits can be maintained). Given that temperament and stress responsiveness are strongly determined by genetics, selection in this direction could be a leverage factor to improve milkability in DCC herds.

## Abbreviations

AMS: automatic milking system
CAM: contact after milking
CBM: contact before milking
CCC: cow-calf contact
DCC: dam-calf contact
HDC: half-day contact
MMY: machine milk yield
OT: oxytocin
WDC: whole-day contact
